# Proteins interaction network and modeling of IGVH mutational status in chronic lymphocytic leukemia

**DOI:** 10.1186/s12976-015-0008-z

**Published:** 2015-06-20

**Authors:** María Camila Álvarez-Silva, Sally Yepes, Maria Mercedes Torres, Andrés Fernando González Barrios

**Affiliations:** Grupo de Diseño de Productos y Procesos (GDPP), Departamento de Ingeniería Química, Universidad de los Andes, Bogotá, DC Colombia; Departamento de Ciencias Biológicas, Facultad de Ciencias, Universidad de los Andes, Bogotá, DC Colombia

**Keywords:** CLL, PPI network, Boolean network, Topological analysis

## Abstract

**Background:**

Chronic lymphocytic leukemia (CLL) is an incurable malignancy of mature B-lymphocytes, characterized as being a heterogeneous disease with variable clinical manifestation and survival. Mutational statuses of rearranged immunoglobulin heavy chain variable (IGVH) genes has been consider one of the most important prognostic factors in CLL, but despite of its proven value to predict the course of the disease, the regulatory programs and biological mechanisms responsible for the differences in clinical behavior are poorly understood.

**Methods:**

In this study, (i) we performed differential gene expression analysis between the IGVH statuses using multiple and independent CLL cohorts in microarrays platforms, based on this information, (ii) we constructed a simplified protein-protein interaction (PPI) network and (iii) investigated its structure and critical genes. This provided the basis to (iv) develop a Boolean model, (v) infer biological regulatory mechanism and (vi) performed perturbation simulations in order to analyze the network in dynamic state.

**Results:**

The result of topological analysis and the Boolean model showed that the transcriptional relationships of IGVH mutational status were determined by specific regulatory proteins (PTEN, FOS, EGR1, TNF, TGFBR3, IFGR2 and LPL). The dynamics of the network was controlled by attractors whose genes were involved in multiple and diverse signaling pathways, which may suggest a variety of mechanisms related with progression occurring over time in the disease. The overexpression of FOS and TNF fixed the fate of the system as they can activate important genes implicated in the regulation of process of adhesion, apoptosis, immune response, cell proliferation and other signaling pathways related with cancer.

**Conclusion:**

The differences in prognosis prediction of the IGVH mutational status are related with several regulatory hubs that determine the dynamic of the system.

**Electronic supplementary material:**

The online version of this article (doi:10.1186/s12976-015-0008-z) contains supplementary material, which is available to authorized users.

## Background

Chronic lymphocytic leukemia (CLL), the most common type of adult leukemia in developed countries, is an incurable malignancy of mature B lymphocytes, characterized by accumulation of mature B cells in the blood, bone marrow, and secondary lymphoid organs such as the lymph nodes (LN) [1, 2]. Patients with CLL show a highly variable disease evolution and different response to therapy. This variability may be related to evolutionary dynamics of sub-clonal mutations [3]. Investigations of the B cell receptor (BCR) indicate that 60–65 % of CLLs carry immunoglobulin heavy chain variable (IGHV) genes with evidence of somatic hypermutation and this may modify BCR affinity for antigens. Conversely, 35–40 % of CLLs are devoid of IGHV somatic mutations [4].

Understanding the pathological mechanisms of CLL has helped to divide the disease into two risk categories that have a strong impact on prognosis and treatment: 1) patients with minimal clinical manifestations and 2) an aggressive form characterized by high mortality, whose IGHV genes can be somatically mutated or unmutated, respectively. Due to the importance of IGVH status in the determination of the course of the disease, several expression studies have focused on the comparison of CLL type mutated IGVH vs. IGVH unmutated. Nevertheless, these studies have identified genes that are not functionally related and therefore cannot elucidate biological mechanisms to distinguish between risk categories.

The interactions of proteins are essential to execute biological functions in different contexts [5]. Since cancer is a complex and multi-factorial disease involving diverse anomalies, the representation and analysis of a malignant cell as a protein-protein interaction (PPI) network can provide insights into its behavior. It has been postulated that proteins with high connectivity within a PPI network could represent meaningful biological information, despite non-being differentially expressed [6]. Thus, the integrated analysis of gene expression data with PPI networks could be valuable method to provide knowledge into molecular mechanisms of diseases. The analyses of PPI networks have varied applications such as identification of drug targets, functional protein modules and disease candidate genes [7, 8].

On the other hand, dynamic network modeling can be used to gain insight into the functionality of biological processed and made possible simulations to predict models behavior [9]. Modeling of regulatory networks as dynamical systems includes modeling based on ordinary differential equations [10, 11], Bayesian framework [12] and Boolean rules [13, 14]. Given the limitation of quantitative models that need knowledge on the kinetics and mechanistic parameters of the system, in addition of a wealth of qualitative and interaction data obtained from the experimental literature and high-throughput technologies, the qualitative approaches such as Boolean modeling become an extremely useful resource [15].

The Boolean modeling considers the genes as binary variables being either active or passive, but encompassing the essential functionality of the system, the general building blocks that have been identified in Boolean networks constitute different types of robust switching elements [16]. This type of approach is already successfully applied in complex models as the FA/BRCA pathway in Fanconi anemia [17], the survival process of in large granular lymphocyte leukemia [18], process of T-helper lymphocytes [19] and the control of the mammalian cell cycle [20].

In this study, we constructed a simplified PPI network of the IGVH mutational status in CLL and analyzed its structure and critical genes. We used the topology of the network to develop a Boolean model, infer regulatory mechanisms and perform simulations to analyze the network in dynamic state. The modeling of the PPI network led to identify regulatory elements of the disease, contributing to understand the prognostic differences and the dynamic behavior under different perturbations over time.

## Results

**Fig. 1:**
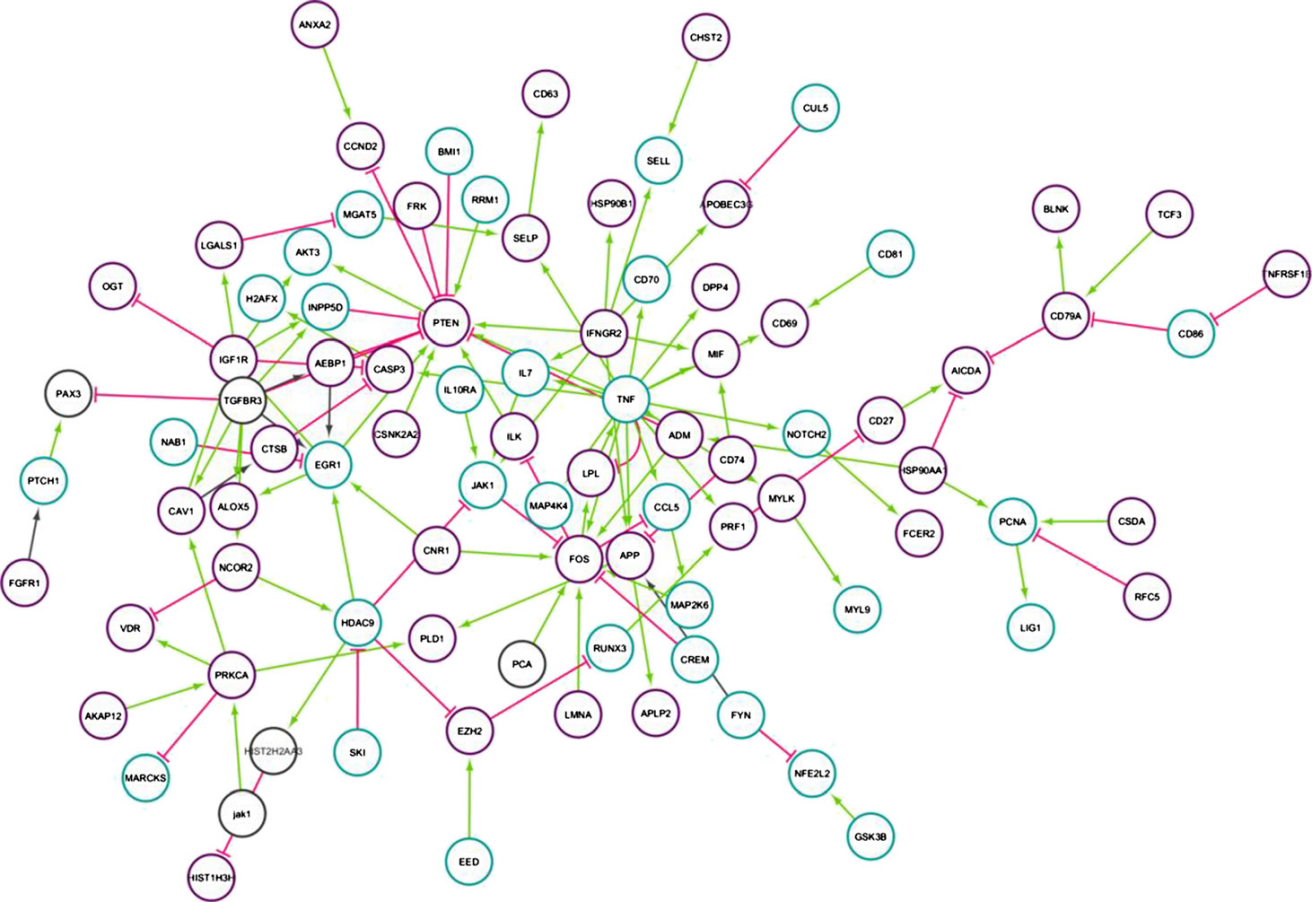
Protein-protein interaction network. Pink edges: inhibition. Green edges: activation. Purple nodes: low expression. Blue nodes: high expression

We inquired into the impact of differentially expressed genes (DEG) between the IGVH statuses by mapping them onto the PPI network. The initial data set of 502 DE genes was reduced to 90 genes with the software STRING and exhaustive literature reviewed. The PPI network reconstructed contains 90 nodes and 120 regulatory edges (Fig. 1). An additional table shows the functions of all genes implicated in the network (Additional file 1: Table S1). The resulting simplified network made evident that DEG between IGVH statuses are not always represented by highly connected nodes.

**Fig. 2:**
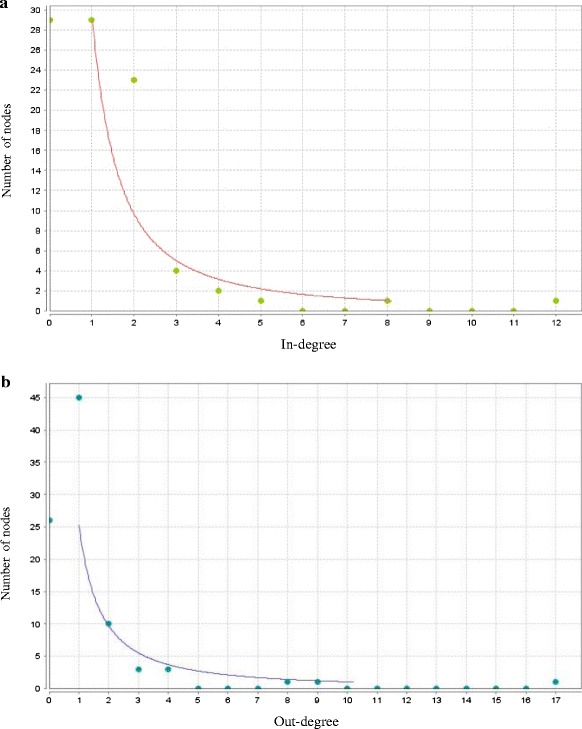
**a**. Power-law in-degree distribution **b**. Power-law out-degree distribution

The main topological characteristic of the network is the degree distribution *P(k)*, which tells us the probability that a select node has exactly *k* links. The degree distribution *P(k)* allows us to distinguish between different types of biological networks. We obtained a power-law degree distribution which characterize scale free networks (Fig. 2), where the probability that a node displays *k* links follows P(k) ∼ k^− γ^, where γ is the degree exponent that describes the role of the hubs in the system [21]. In the PPI network we obtained values of γ between 2 and 3, indicating that there exist a hierarchy of hubs, i.e. there are a large number of nodes with few connections while highly connected nodes are scarce [21]. The following genes showed the highest values in degree evaluation: in-degree (PTEN, FOS, and EGR1) and out-degree (TNF, TGFBR3, and IFGR2). Values of γ between 2 and 3 refer also to the small-world property [22], characterized by a small value of diameter, which increase the network efficiency. However, we obtained a value of diameter of 8, greater-than-expected for networks with the small-world property. To explain the values of diameter higher to the expected, Zhang *et al*. [22] made a comparison between the values of diameter reported for real biological networks regarding to diameters obtained for networks with the same features in which the connections between nodes are randomized. Specifically, they evaluated the diameter of protein-protein interaction networks of biological organisms in contrast to the obtained in randomized networks. Their simulation showed that an increment of the diameter in the real networks allows a significant increase of the network modularity, suggesting an adjustment between network efficiency and the benefits obtainable by modularity [22].

**Fig. 3:**
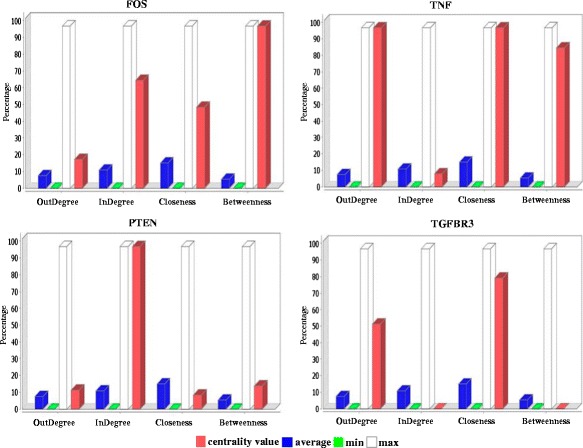
Measure of centralities of FOS, TNF, PTEN and TGFBR3

This analysis was completed by other centralities measures such as closeness and betweenness, which provide a characterization of nodes that are relevant for the network structure. Closeness is defined by the inverse of the average length of the shortest paths to all other nodes [23]. We found TNF, TGFBR3, and IFGR2 as proteins with the highest closeness values in the PPI network. A protein with high closeness, compared to the average closeness of the network, will be easily central to the regulation of other proteins but with some proteins not influenced by its activity [24]. Implying that those proteins are the closest to all other nodes and have an extent of influences on the entire network. This parameter can also be considered a measure of how long it will take information to spread from a given node to others [25]. Betweenness is defined by the number of shortest paths that pass through a node [26]. The betweenness index favors nodes that join communities rather than nodes that lie inside a community [27, 28]. FOS, TNF and LPL exhibited the highest values, implying a role as linkers in the control of interactions between proteins. Selected genes, reported above, can be seen in Fig. 3. The overall parameters that characterized the network are shown in the Additional file 2: Table S2, and the topological parameters for each one of the nodes are shown in the Additional file 3: Table S3.

We saw that centrality measures in the PPI network shown some degrees of overlap, stressing the importance of the implicated proteins in the system structure. According to Yu *et al.* [29], as a complementary notion of highly connected proteins known as hubs proteins, it is possible to define bottlenecks proteins as proteins with high betweenness values, bottlenecks proteins are essential connectors with surprising functional and dynamic properties. Therefore, to develop a Boolean model and evaluate the genes with influence in the behavior of the network over time, we focused on those that were at the center of the major structural hubs and simultaneously exhibited the highest values in the topological centralities evaluated. The selected nodes were: FOS, PTEN, TGFBR3, and TNF. The dependencies between the genes obtained from the literature review were translated to rule sets. The biological events for activation or inhibition were qualitatively represented by Boolean functions, that is, combinations of AND, OR, and NOT operations, that determine the evolution of a node through time and their relation to the other components of the system (Additional file 4: Table S4).

**Fig. 4:**
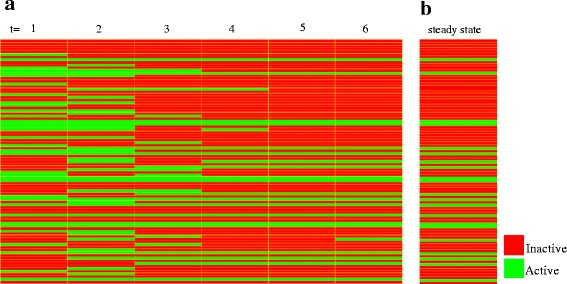
Visualization of a sequence of states. **a.** The columns of the table represent consecutive states of the time series. **b**. Steady-state attractor of the network from initial state determined by microarray analysis. Genes are encoded in the following order: AEBP1 AFF1 AICDA AKAP12 AKT3 ALOX5 ANXA2 APLP2 APOBEC3G APP BLNK BMI1 CASP3 CAV1 CCL5 CCND2 CD27 CD63 CD69 CD70 CD79A CD81 CD86 CHST2 CNR1 CREM CSDA CSNK2A2 CTSB CUL5 DPP4 EED EGR1 EZH2 FCER2 FGFR1 FOS FRK FYN GSK3B H2AFX HDAC9 HIST1H3H HIST2H2AA3 HSP90AA1 HSP90B1 IFNGR2 IGF1R IL10RA IL7 ILK INPP5D JAK1 LGALS1 LIG1 LMNA LPL MAP2K6 MAP4K4 MARCKS MGAT5 MIF MYL9 MYLK NAB1 NCOR2 NFE2L2 NOTCH2 OGT PAX3 PCNA PLD1 PRF1 PRKCA PTCH1 PTEN RFC5 RPS6KA5 RRM1 RUNX3 SELL SELP SIAH1 SKI TCF3 TNF TNFRSF1B VDR CD74 ADM TGFBR3. Active genes in this attractor state were: AFF1, APLP2, APP, BMI1, CD27, CD81, CD86, CREM, CUL5, EED, EZH2, FYN, GSK3B, HIST2H2AA3, HSP90B1, IL10RA, ILK, MARCKS, MGAT5, RRM1, SKI

**Fig. 5:**
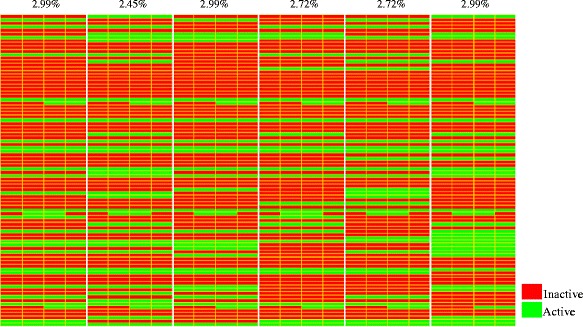
Major attractors obtained from 50 random initial states. The columns of the table represent consecutive states of the attractor. On top, the percentage of states leading to the attractor is supplied. Genes are encoded in the following order: AEBP1 AFF1 AICDA AKAP12 AKT3 ALOX5 ANXA2 APLP2 APOBEC3G APP BLNK BMI1 CASP3 CAV1 CCL5 CCND2 CD27 CD63 CD69 CD70 CD79A CD81 CD86 CHST2 CNR1 CREM CSDA CSNK2A2 CTSB CUL5 DPP4 EED EGR1 EZH2 FCER2 FGFR1 FOS FRK FYN GSK3B H2AFX HDAC9 HIST1H3H HIST2H2AA3 HSP90AA1 HSP90B1 IFNGR2 IGF1R IL10RA IL7 ILK INPP5D JAK1 LGALS1 LIG1 LMNA LPL MAP2K6 MAP4K4 MARCKS MGAT5 MIF MYL9 MYLK NAB1 NCOR2 NFE2L2 NOTCH2 OGT PAX3 PCNA PLD1 PRF1 PRKCA PTCH1 PTEN RFC5 RPS6KA5 RRM1 RUNX3 SELL SELP SIAH1 SKI TCF3 TNF TNFRSF1B VDR CD74 ADM TGFBR3

Starting from an initial condition, the Boolean model evolved over time to finally stabilizes in a recurrent state known as attractor, representing the long-term behavior of the system [15]. We found a simple attractor for the PPI network for CLL consisting of one state. The model obtained achieves the fixed point (steady state) after six time steps. The state of transition and its successor attractor (starting from the initial state determined by microarray analysis) are shown in Fig. 4. Starting from 50 random initial states, the system had both the single-state attractors and cycle attractors. The major cycle attractors displayed four states (Fig. 5). This showed important dependency of the achieved attractor according to the initial system state.

Given the relevance of signaling pathways as triggers of processes associated with cancer oncogenesis and progression, the activated proteins in the attractor were subjected to modular functional enrichment to determine annotations from KEGG and Panther pathways simultaneously. The associated annotations involved various pathways related to cancer with statistical significance for focal adhesion (corrected *p* value = 0.000231747). Other signaling pathways detected in the attractor included: B cell receptor, T cell receptor, cytokine-cytokine, integrin, and cadherin, among others. Processes related with cell proliferation and regulations of transcription were also present.

Given the overriding importance attributed to FOS and TNF in cancer biology and their high topological measures in the PPI network, we chose these genes to performed perturbation simulations of knockout and overexpression in the system. Furthermore, we analyzed the effect of different initial conditions that can lead the system to different steady states (attractors). The two initial conditions that were taken into account were: i) initial condition determined by microarray analysis and ii) an initial state in which all nodes were active.

The overexpression of FOS produced activation of genes related with multiples signaling pathways. When comparing activated genes under the FOS overexpression regardless of activated genes in the original attractor, the modular functional analysis found the MAPK signaling pathway with the most significant value, (*p* = 4.37211e-05). Other pathways involved were: apoptosis (*p* = 7.34148e-05), PDGF (*p* = 0.000100226), JAK-STAT (*p* = 0.0001216), angiogenesis (*p* = 0.0001216) and cytokine-cytokine interaction (*p* = 0.000500178). Similarly, activated genes under TNF overexpression were involved in multiple signaling pathways associated with cancer: focal adhesion (*p* = 7.05488e-07), angiogenesis (*p* = 8.39556e-07), JAK-STAT (*p* = 1.39118e-06), tight junction (*p* = 5.76719e-06), MAPK (*p* = 7.60619e-06), Wnt (*p* = 0.000105245), among others.

Under both initial conditions the effect of knocking out of any gene did not display an effect, since the same attractor was obtained when no gene was knocked out. Nevertheless, the path length to achieve the attractor varies significantly, that is, depending on the initial conditions, the system takes more or less steps of evolution in time to stabilize in a recurrent state, known as attractor. From this behavior of the system, it can be concluded that the attractor achieved is the most stable state of the network, because although there are perturbations involving the system, the network returns to the same steady state after different steps of time evolution.

On the other hand, when constantly maintaining the major nodes active under the two initial conditions studied, we found that the selected gene displayed an effect on the attractor achieved and therefore on the behavior of the system, affecting evolution of the network. These changes in the behavior are important to determine its relation to the evolution of the disease.

**Fig. 6:**
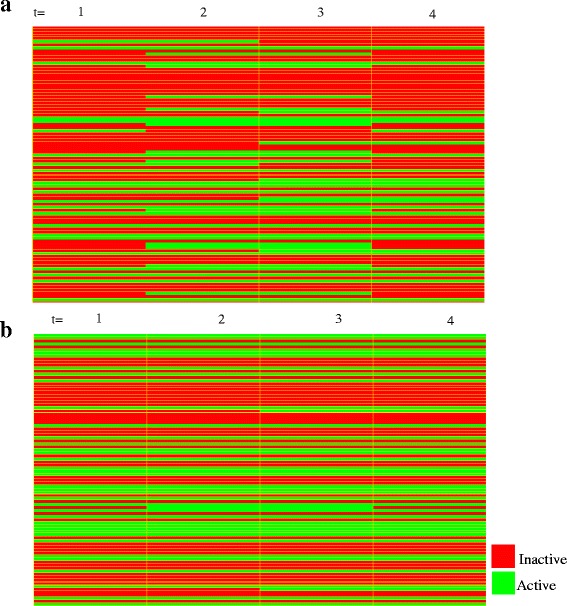
Genes are encoded in the following order: AEBP1 AFF1 AICDA AKAP12 AKT3 ALOX5 ANXA2 APLP2 APOBEC3G APP BLNK BMI1 CASP3 CAV1 CCL5 CCND2 CD27 CD63 CD69 CD70 CD79A CD81 CD86 CHST2 CNR1 CREM CSDA CSNK2A2 CTSB CUL5 DPP4 EED EGR1 EZH2 FCER2 FGFR1 FOS FRK FYN GSK3B H2AFX HDAC9 HIST1H3H HIST2H2AA3 HSP90AA1 HSP90B1 IFNGR2 IGF1R IL10RA IL7 ILK INPP5D JAK1 LGALS1 LIG1 LMNA LPL MAP2K6 MAP4K4 MARCKS MGAT5 MIF MYL9 MYLK NAB1 NCOR2 NFE2L2 NOTCH2 OGT PAX3 PCNA PLD1 PRF1 PRKCA PTCH1 PTEN RFC5 RPS6KA5 RRM1 RUNX3 SELL SELP SIAH1 SKI TCF3 TNF TNFRSF1B VDR CD74 ADM TGFBR3. **a**. Visualization of states under overexpression of FOS **b**. Visualization of states under overexpression of TNF

Under both conditions of perturbation, the overexpression of the gene FOS and TNF showed important influence in the evolution over time of the system (Fig. 6). In both cases the system reaches complex attractors in which the network oscillates among a set of four states, i.e., the attractor of the network is a cycle. In these states the nodes involved in regulation of CLL oscillate under states of OFF or ON, which affects the course of the disease.

## Discussion

We applied a system approach by linking proteins interaction data with differentially expressed genes of the IGVH status, the reconstructed PPI network allowed identified critical genes, and given the stringent parameters applied, they represent only relationships based on strong protein interactions. The topology of the reconstructed PPI network followed the power-law of node degree distribution, a feature of true complex biological networks. Therefore, the obtained network is a scale-free biological entity rather than a random network, indicating the presence of few nodes having a very high degree measure [21]. On the other hand, it is recognized that high values of degree centrality are associated with proteins that are interacting with several others suggesting a central regulatory role [23]. The degree index highlighted some important proteins with regulatory functions and considered interactions hubs in the PPI network: PTEN, FOS, EGR1, TNF, TGFBR3, and IFGR2. According to the lethality and centrality rule, the highly connected nodes are biologically relevant, representing vulnerable and essential points to system viability [30, 31], supporting this argument the establishment and stability of cancer cells through these hubs.

It was noted that highly connected proteins in the PPI network are not necessarily represented for highly DEG. Consequently, genes with a central role in cancer not detected for high-throughput approaches could be identified by networks based analysis [6]. This was the case for PTEN, FOS, EGR1 and TNF, whose p values were significant but they were not among the lowest in the meta-analysis. Several genes with known prognostic implications in CLL were present in the list of DEG; additionally, the top DEG obtained were consistent with other studies [32–34], validating these findings the microarray meta-analysis approach to produce robust conclusions.

LPL is one for the strongest prognostic markers to predict outcome in CLL [35, 36]. It was one of the genes with high betweenness index, reflecting the large amount of control exerted by this node over the interactions between the other nodes, in this way, LPL may function as bridge between sub-graphs. According to Kolset and Salmivirta [37] LPL facilitate the contact between monocytes and endothelial cell through its union with heparan sulfate proteoglycans, serving as a bridging protein between cell surface proteins and lipoproteins. On the other hand, LPL may influence CLL behavior for its relation with functional pathways involved in fatty acid degradation and signaling [38].

All genes found with high centralities have central roles in cancer and are involved in major, diverse and sometimes interrelated signaling pathways. They have roles as tumor suppressors or oncogenes, engaging central role in cancer progression. PTEN has phosphatase catalytic function that antagonizes the PI3K/AKT signaling pathway and suppresses cell survival as well as cell proliferation [39]. TNF gene encodes a multifunctional proinflammatory cytokine that belongs to the tumor necrosis factor (TNF) superfamily, can induce a wide range of intracellular signal pathways including apoptosis and cell survival as well as inflammation and immunity. TNF has two receptors (TNFR1, TNFR2), TNFR1 signaling induces activation of many genes, primarily controlled by two distinct pathways, NF-kappa B pathway and the MAPK cascade, or apoptosis and necrosis. TNFR2 signaling activates NF-kappa B pathway, including PI3K-dependent NF-kappa B pathway and JNK pathway leading to survival [40]. FOS gene family encodes leucine zipper proteins that can dimerize with proteins of the JUN family, thereby forming the transcription factor complex AP-1. As such, the FOS proteins have been implicated as regulators of cell proliferation, differentiation, and transformation. In some cases, expression of the FOS gene has also been associated with apoptotic cell death [41]. Studies about EGR1 have been suggested that it is a cancer suppressor gene and a transcriptional regulator. The products of target genes it activates are required for differentiation and mitogenesis [42]. TGFBR3 is a membrane proteoglycan that functions as a co-receptor with other transforming growth factors receptors. Soluble TGFBR3 may inhibit TGFB signaling. Decreased expression of this receptor has been observed in various cancers [41].

When the attractors found in the analysis of Boolean model are analyzed, several key proteins associated with specific signaling pathways related with cancer are found, it became clear that the phenotype depends upon multiple and interrelated signaling pathways. We underscore the importance of MAPK signaling pathway, identified by enrichment analysis, under FOS overexpression in the Boolean model. Belonging to the MAPK/ERK signaling cascade were found activated: CASP3, FGFR1, AKT3, FOS, MAP2K6, MAP4K4, PRKCA, RPS6KA5, and TNF. Aberrations in the MAPK/ERK pathway have been identified in human cancers in high frequency including hematologic malignancies [42]. In the context of CLL, the MAPK signaling pathway has been recently implied in the disease based on clustering of RNA sequencing data [43]. Similarly, working with gene co-expression subnetworks associated with disease progression, it has been proven association of MAPK pathway with higher expression levels in patients at early stages of the disease [44].

The regulatory hubs determine the behavior of the disease over time. The dynamics of the network is controlled by attractors involved of diverse signaling pathways, which may suggest a variety of mechanisms controlling the difference in CLL behavior. “The 2 distinct disease” hypothesis in CLL could be challenged; it is an interesting approach to speculate that the CLL disease transcriptome evolves over time to reach a state associated with disease requiring treatment [44]. These results have implications for understanding transcriptional dynamic in the evolution of the disease.

## Conclusion

The PPI network and a Boolean model of IGVH mutational status in CLL allowed identified regulatory proteins and generated insight about processes associated with the manifest differences in prognosis. The perturbation in the network through overexpression of important regulatory proteins, such as FOS and TNF, determine the dynamic of the network and activate genes involved in different signaling pathways that play important roles in cancer.

## Methods

### Differential expression analysis between the IGVH mutational statuses

To ensure reliability and generalization of results, we combined information from different and independent microarray expression cohorts. It is well known that integration of expression data allow the discovery of new biological insights by increasing the statistical power [45]. We retrieved CLL cohorts from the Gene Expression Omnibus (GEO) of the National Center for Biotechnology Information (NCBI). The cohorts selected (GSE2466, GSE16746, GSE9992 and GSE38611) had raw data available, were originally processed with different microarray platforms and had at least 60 CLL patients with information about the IGVH statuses, in total were processed 356 CLL patients (174 mutated/umutated status). Each study was normalized independently using the VSN method implemented in R [46]. For filtering non-expressed and non-informative genes, matching genes among different microarray platforms and merging among studies, we used MetaDE package in R [47]. To combining the information of cohorts and avoid batch effect we followed a meta-analysis approach, the moderated-t statstics with permutations was used for individual analysis and Fisher *P*-value combination method for combine the individual p values [48]. For meta-analysis we used the “MetaOmics” software suite [47].

### Reconstruction of protein–protein interaction network

The reconstruction of the protein–protein interaction network was based on data from differential expression analysis between IGVH mutational statuses. The list of 502 DE genes obtained was used to retrieve interacting partners from curated databases and current literature.

The protein–protein interaction network was constructed based on the current literature and, through the STRING—Search Tool for the Retrieval of Interacting Genes/Proteins—web source [49]. The STRING database contains information from several sources, including experimental data, computational prediction methods, public text collection and an recompilation of predicted protein interactions of databases such as EXPASY [50], BIND [51], BioGRID [52], DIP [53], IntAct MINT [54], and HPRD [55] and with interactions from the pathway databases such as PID [56], Reactome [57], KEGG [58], and EcoCyc [59].

In order to reduce the amount of data while maintaining the main gene relations, the parameters of confidence for STRING were restricted for obtain more reliable associations. Furthermore, the active prediction methods taken into account for STRING predictions were: co-expression, experiments, databases, and text mining. From the protein-protein interactions predicted by STRING, with the restrictions mentioned above, only the genetic relationships causing the activation or inhibition of the components of the network were considered. In this way we reduced the number of network nodes from 502 to 90. Is important to state that the database predictive methods could reduce the overall confidence of network.

### Network topology analysis

Topological analysis of the protein–protein interaction network was carried out by plugin Network Analysis [60] of the open source program Cytoscape 3.1.0 [61].

A topological evaluation of the network was carried out by evaluating structural parameters such as the clustering coefficient and degree distributions, to evaluate the number of interactions among one node and its neighbors, normalized by the maximum number of possible interactions, and to determine the number of nodes directly connected (first neighbors) to a given node *v*, respectively. In the network evaluation we distinguished in-degree distribution, when the edges target the node *v*, and out-degree distribution, when the edges target the adjacent neighbors of *v* [62, 60].

The evaluation of the relevance of a protein in the PPI network was also made through measurements of network centrality parameters, for this purpose, were used the betweenness, closeness and degree metrics. On the other hand, to analyze the “compactness” of the network, we evaluate the average path length and the network diameter, parameters that indicate how distant are the two most distant nodes, showing the overall proximity between nodes in the interaction network analyzed [21].

### Boolean network model

In a Boolean network model, each node *i* = 1, 2, …, *N* represents a protein of the network that can assume only binary states θ_i_. When θ_i_ = 1 the protein is functionally active (TRUE), on the other hand, when θ_i_ = 0 the protein is functionally inactive (FALSE). Thus, a network with N nodes will have 2 N possible states [21]. In this model, edges represent regulatory relationships between elements; their orientation in the network follows the direction of regulation process from the upstream to the downstream node. As time passes, the state of each node is determined by the states of its neighbor, through Boolean transfer functions based on evidence from the literature [63, 64].

The exponential behavior of the possible states in a Boolean network makes it computationally unsuitable for large networks, where it is necessary to reduce the network size, restricting the protein–protein interactions to the relationships of activation or inhibition of any of the components of the system.

In this study, the Boolean synchronous algorithm proposed by Stuart Alan Kauffman in 1969 [13] was implemented. The synchronous pattern is the most simple update mode, where the states of all nodes are updated simultaneously according to the last state of the network [15]. We used the package BoolNet [65] to construct Boolean synchronous networks from knowledge of the dependencies of genes, based on evidence from the current literature.

Starting from an initial condition, the model evolves over time to finally stabilize in a recurrent state known as attractor, representing the long-term behavior of the system [15]. Different initial conditions for the model may lead the system to different attractors, whereby the Boolean model should start from previously known biological information; in this model the initial states of the network were determined from results supplied for microarray analysis of up and down regulation states (Additional file 4: Table S4). Aiming to prove the sensitivity of the Boolean model, we assayed 50 random initial states, and demonstrated that the constructed model shows changes in the structure of the attractor achieved in the steady state under different initial states.

To determine critical proteins for structure and behavior of the system, we examined the changes in network attractors if a certain component is knocked out (fixed in the OFF state in the Boolean model) or overexpressed (fixed in the ON state in the Boolean model). If knocking out or overexpressing a component leads to changes in network dynamic response, it can be concluded that this component is implicated in the biological regulation processes [15].

### Functional enrichment analysis

Significant concurrent annotations from KEGG and Panther pathways were searched with GeneCodis software [66–68].

## Additional files

Additional file 1: Table S1. Summary of genes functions. Additional file 2: Table S2. Topological properties of the CLL network. Additional file 3: Table S3. Topological parameters for the individual nodes. Additional file 4: Table S4. Boolean transfer functions and initial conditions of the Boolean network.

## Abbreviations

CLL: Chronic lymphocytic leukemia
IGVH: Immunoglobulin heavy chain variable
PPI: Protein-protein interaction
DEG: Differentially expressed genes
